# Measuring Internal Maturity Parameters Contactless on Intact Table Grape Bunches Using NIR Spectroscopy

**DOI:** 10.3389/fpls.2019.01517

**Published:** 2019-11-28

**Authors:** Andries J. Daniels, Carlos Poblete-Echeverría, Umezuruike L. Opara, Hélène H. Nieuwoudt

**Affiliations:** ^1^Department of Viticulture and Oenology, Faculty of AgriSciences, Stellenbosch University, Stellenbosch, South Africa; ^2^Crop Development Department, ARC Infruitec-Nietvoorbij, Private bag X5026, Stellenbosch, South Africa; ^3^Postharvest Technology Research Laboratory, South African Research Chair in Postharvest Technology, Department of Horticultural Sciences, Faculty of AgriSciences, Stellenbosch University, Private bag X1, Stellenbosch, South Africa; ^4^Institute for Wine Biotechnology, Department of Viticulture and Oenology, University of Stellenbosch, Private bag X1, Stellenbosch, South Africa

**Keywords:** table grapes, near-infrared spectroscopy, total soluble solids, titratable acidity, BrimA

## Abstract

The determination of internal maturity parameters of table grape is usually done destructively using manual methods that are time-consuming. The possibility was investigated to determine whether key fruit attributes, namely, total soluble solids (TSS); titratable acidity (TA), TSS/TA, pH, and BrimA (TSS – *k* x TA) could be determined on intact table grape bunches using Fourier transform near-infrared (FT-NIR) spectroscopy and a contactless measurement mode. Partial Least Squares (PLS) regression models were developed for the maturity and sensory quality parameters using grapes obtained from two consecutive harvest seasons. Statistical indicators used to evaluate the models were the number of latent variables (LVs) used to build the model, the prediction correlation coefficient (R^2^p) and root mean square error of prediction (RMSEP). For the respective parameters TSS, TA, TSS/TA, pH, and BrimA, the LVs were 21, 23, 5, 7, and 24, the R^2^p = 0.71, 0.33, 0.57, 0.28, and 0.77, and the RMSEP = 1.52, 1.09, 7.83, 0.14, and 1.80. TSS performed best when moving smoothing windows (MSW) + multiplicative scatter correction (MSC) was used as spectral pre-processing technique, TA with standard normal variate (SNV), TSS/TA with Savitzky-Golay first derivative (SG1d), pH with SG1d, and BrimA with MSC. This study provides the first steps towards a completely nondestructive and contactless determination of internal maturity parameters of intact table grape bunches.

## Introduction

The logistics of table grape harvest and shipment to intended consumer markets is complex and challenging. Table grapes (*Vitis vinifera* L.) is a nonclimacteric fruit, which does not ripen further, nor does the quality improve after harvest (Sonego et al., 2002). Therefore, grapes must be at the desired maturity level when harvested and the eating quality of packed produce must be retained during several weeks of cold storage and ultimate shipment to markets. Traditionally, fruit maturity is expressed in terms of total soluble solids (TSS), also referred to as soluble solids content (SSC), which primarily reflects the sugar content, and titratable acidity (TA), which reflects the tartaric acid content (Nelson et al., 1963). Although pH is usually included as part of the routine chemical analysis to assess the maturity and sensory characteristics of grapes, no clear link has yet been established between pH and grape maturity (Walker et al., 2001; Reynolds et al., 2006). TSS is typically measured in the vineyard with a handheld refractometer and expressed as °Brix, while TA is determined in the laboratory by wet chemistry methods. Worldwide, TSS and sugar/acid ratios (TSS/TA) serve as primary indices for the quality of export fruit. Minimum requirements are specified for TSS concentrations and TSS/TA ratios for each cultivar, for example by the Agricultural Product Standards Act, 1990 (Act No. 119 of 1990) of South Africa, section 4(3)(a)(ii). Harvested table grape bunches are packed and exported either as individual bunches in punnets, or individually wrapped and packed in a box together with other bunches. When table grape consignments reach the harbour of the exporting country, random spot checks are done on packed fruit. If any sample is found to be at the incorrect TSS and/or TSS/TA ratio, whole export consignments can be rejected, or even returned once they have reached the intended market. Given that the popularity of table grapes makes it one of the most consumed fruits in the world (Piazolla et al., 2013), anything that affects quality negatively and leads to losses should be avoided.

All the aforementioned laboratory measurements are done destructively and are time consuming. Furthermore, measurement of TA requires both specialised equipment and chemicals and creates chemical waste. Opportunities for the table grape industry to move away from destructive techniques to determine key maturity parameters (TSS, TA, TSS/TA, and pH) already exist. Fourier transform near infrared (FT-NIR) spectroscopy has long been used with success to determine a wide variety of parameters in fruit. Nondestructive postharvest determination of TSS, TA, and pH have been reported on apricots (Camps and Christen, 2009), pears (Liu et al., 2008), mandarins (Liu et al., 2010), plums (Pérez-Marín et al., 2010), blueberries (Sugiyama et al., 2010), avocados (Wedding et al., 2010), wine grapes (González-Caballero et al., 2010; Kemps et al., 2010; Barnaba et al., 2013), and individual table grape berries (Cao et al., 2010; Omar, 2013).

Challenges related to quality evaluation of intact bunches include the complexity of their morphology which includes the number of berries on the bunch and, the shape and compactness of the bunch (Mattheou et al., 1995; May, 2000), which in turn have been shown to be dependent on the grape cultivar (Balic et al., 2014). Other factors which add to the challenge of scanning intact bunches include the within-bunch and between-bunch heterogeneity in sugar and maturity levels (Mattheou et al., 1995; Šuklje et al. (2012). These aspects are known to be influenced by the seasonal effects as well as the geographical location of the vineyards (Sonego et al., 2002). The double sigmoidal growth curve associated with grape development and ripening stages has been thoroughly discussed by several authors (Dokoozlian and Kliewer, 1996; Wheeler et al., 2009) and recently on table grapes by Sonnekus (2015). It is, however, important to emphasize the complex role temperature plays in the ripening (Kuhn et al., 2014) and hence quality of grapes (Coombe, 1987). Fluctuations in the maximum and minimum temperatures during consecutive seasons can lead to grapes either ripening earlier or later than might be anticipated. This has serious consequences on the marketability of table grapes for the producers.

In this study, the potential of NIR spectroscopy to quantify TSS, TA, TSS/TA, and pH nondestructively on intact bunches is explored. Individual bunches were scanned contactless using diffuse reflectance FT-NIR spectroscopy. To enrich the information gathered on the mentioned quality parameters, another sensory-based parameter, namely, BrimA (calculated as TSS - *k* x TA), and originally proposed by (Jordan et al., 2001), was also included in the analysis. BrimA is an alternative parameter for determining the palatability of table grapes. Jordan et al. (2001) argued that the TSS/TA ratio does not fully reflect the major influence that acid has on the tastiness prediction of table grapes. The human tongue does not have the same sensitivity for sugar than it has for acidity. However, Jayasena and Cameron (2008), argued that TSS/TA ratio is a better indicator of consumers’ taste acceptance of Crimson Seedless table grapes than TSS, TA, and BrimA alone. Fawole and Opara (2003) also reported that both the TSS/TA ratio and BrimA are useful to create a dependable index for evaluating optimal fruit maturity of pomegranates. The inclusion of both TSS/TA ratio and BrimA as sensory parameters in this study was, therefore, of utmost importance to pave the way for nondestructive evaluation of the taste acceptability of grapes. To our knowledge, this is the first report on analysis of completely intact bunches using FT-NIR spectroscopy.

## Materials and Strategies

### Grape Sampling

**Figure 1:**
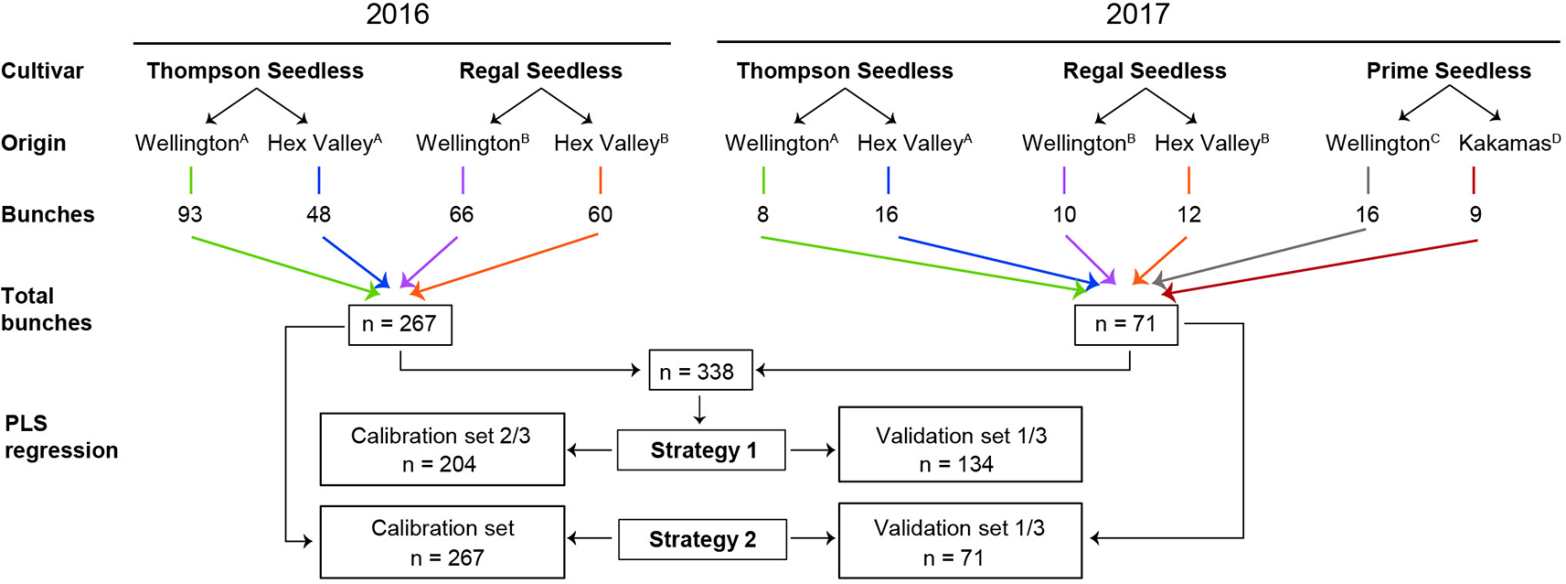
Experimental design for the 338 intact table grape bunches subjected to Fourier transform near-infrared (FT-NIR) spectroscopy. ^A^ Grapes harvested from the same vineyard block in both years; ^B^ Grapes harvested from the same vineyard block in both years; and then ^C,D^ Grapes harvested from these two new vineyards blocks in 2017.

The experimental design in **Figure 1** shows the harvest years, cultivars, location of the vineyard cultivars were harvested from, number of bunches harvested per location and per year, as well as the two strategies followed to build PLS models for the parameters under investigation. Our experimental design aimed to include variability resulting from seasonal effects, vineyard geographic location, ripeness levels, and grape cultivar. Grapes were harvested from three locations over two seasons (2016 and 2017), and at two ripening stages. Three white seedless table grape cultivars were used, i.e., Prime Seedless, Thompson Seedless, and Regal Seedless, which are amongst the top 20 cultivars exported from South Africa (SATI, 2018).

**Table 1** shows the GPS co-ordinates, harvest week, and the TSS level for the three cultivars at the individual locations. Grapes were harvested from the fifth row of each block starting from the third section of the row. The vines were marked so that grapes could be harvested from the same vines in the two consecutive years. The rationale for this step was to reduce the number of factors that would play a role in each year. Soils, as well as the microclimate which influences the development of bunches (accumulation of sugar and breakdown of acids) may vary within a block (Šuklje et al., 2012). Bunches were randomly selected from the vines on both sides of the canopy and each cultivar was harvested twice on two separate dates from each location. The respective distances from the vineyards to the laboratory in Stellenbosch are Kakamas 840 km, Wellington 42 km, and Hex Valley 139 km. Grapes were harvested and packed in the morning before 10 h and kept at 20°C during transport to the laboratory. A total of 338 grape bunches was scanned on the infrared spectrometer within twelve hours after harvest.

**Table 1:** GPS co-ordinates, harvest week, and TSS level of grapes.

Cultivar	Site	Latitude	Longitude	Altitude	2016 Harvest Week	2017 Harvest Week	2016 TSS^d^	2017 TSS
Thompson Seedless	HV^a^	33°27’53,9”S	19°39’43,7”S	907 m	W3	W4	16.85	15.64
					W4	W5	Stolen	16.62
Thompson Seedless	W^b^	33°37’03,5”S	18°58’05,3”S	904 m	W3	W3	17.49	18.72
					W4	W5	18.62	Rotten
Regal Seedless	HV	33°27’50,4”S	19°39’47,6”E	904 m	W3	W5	18.39	19.41
					W5	W5	21.27	21.36
Regal Seedless	W	33°30’14,2”S	10°50’40,0”E	904 m	W3	W4	15.47	14.12
					W5	W6	16.44	16.34
Prime Seedless	W	33°38’22,0”S	10°50’47,6”E	900 m	W51		10.65	
					W52		12.02	
Prime Seedless	K^c^	28°37’54,8”S	20°26’38,6”E	903 m	W48		14.89	
					W50		16.08	

^a^Hex Valley; ^b^Wellington; ^c^Kakamas;^d^Total soluble solids in °Brix measured with a handheld refractometer.

**Table 2** shows the lowest, highest, and average daily temperatures for the different locations taken from weather stations in the nearest vicinity of the blocks from which grapes were harvested from during the two seasons. These weather stations were Hex Valley PP with latitude = -33,46609, longitude = 19,66304 and altitude = 459 for Hex Valley; Eureka with latitude = -33,69301, longitude = 18,95259 and altitude = 161 for Wellington and Kromhout Boerdery with Latitude = -28,7869, Longitude 18,95259 and Altitude = 161 for Kakamas. The values in bold indicate where the daily average maximum and minimum temperatures were higher in the second season and the underlined values indicate where the daily average maximum and minimum temperatures were lower in the second season. The influence this had on the maturity and sensory parameters will be discussed further down in the manuscript.

**Table 2:** Temperature data for the sites from which the grapes were harvested.

Site	Month	Day	Tx^a^	Tn^b^	Tx	Tn	Tx	Tn
			2015		2016		**2017**	
HV^d^	11	Lowest	19.75^c^	6.38	21.83	3.32		
HV	11	Highest	37.64	22.65	37.67	16.27		
HV	11	Average	27.18	17.8	**28.88**	9.21		
HV	12	Lowest	26.63	19.9	25.29	8.83		
HV	12	Highest	35.37	28.42	39.99	16.38		
HV	12	Average	30.23	23.65	**33.01**	11.92		
HV	1	Lowest			33.36	26.1	24.5	8.62
HV	1	Highest			33.55	28.31	38.24	17.88
HV	1	Average			33.46	27.45	32.09	12,66
HV	2	Lowest			26.02	8.13	27.97	7.66
HV	2	Highest			39.91	19.6	38.02	18.15
HV	2	Average			31.46	12.29	**33.29**	**12.85**
W^e^	11	Lowest	17.55	8.02	20	9.55		
W	11	Highest	38.66	20.05	35.54	20.05		
W	11	Average	27.34	13.73	**28.44**	**14.58**		
W	12	Lowest	23	12.44	21.41	12.6		
W	12	Highest	41.09	22.34	36.99	19.87		
W	12	Average	30.94	16.46	**31.22**	15.67		
W	1	Lowest			24.26	15.82	23.35	12.91
W	1	Highest			39.97	25.3	38.34	20.48
W	1	Average			33.99	20.92	31.37	16.56
W	2	Lowest			25.14	12.5	24	14.08
W	2	Highest			38.41	24.52	40	24.64
W	2	Average			31.49	17.53	**32.3**	**17.87**
K^f^	11	Lowest			30.25	8.9		
K	11	Highest			41.14	21.33		
K	11	Average			36.15	14.37		
K	12	Lowest			33.18	11.72		
K	12	Highest			44.05	22.76		
K	12	Average			38.71	16.97		

^a^Daily Maximum Temperature; ^b^Daily Minimum Temperature, ^c^Unit = °C; ^d^Hex Valley; ^e^Wellington; ^f^Kakamas. The values in bold indicate where the daily average maximum and minimum temperatures were higher in the second season.

### Fourier Transform Near-Infrared Spectroscopy

**Figure 2:**
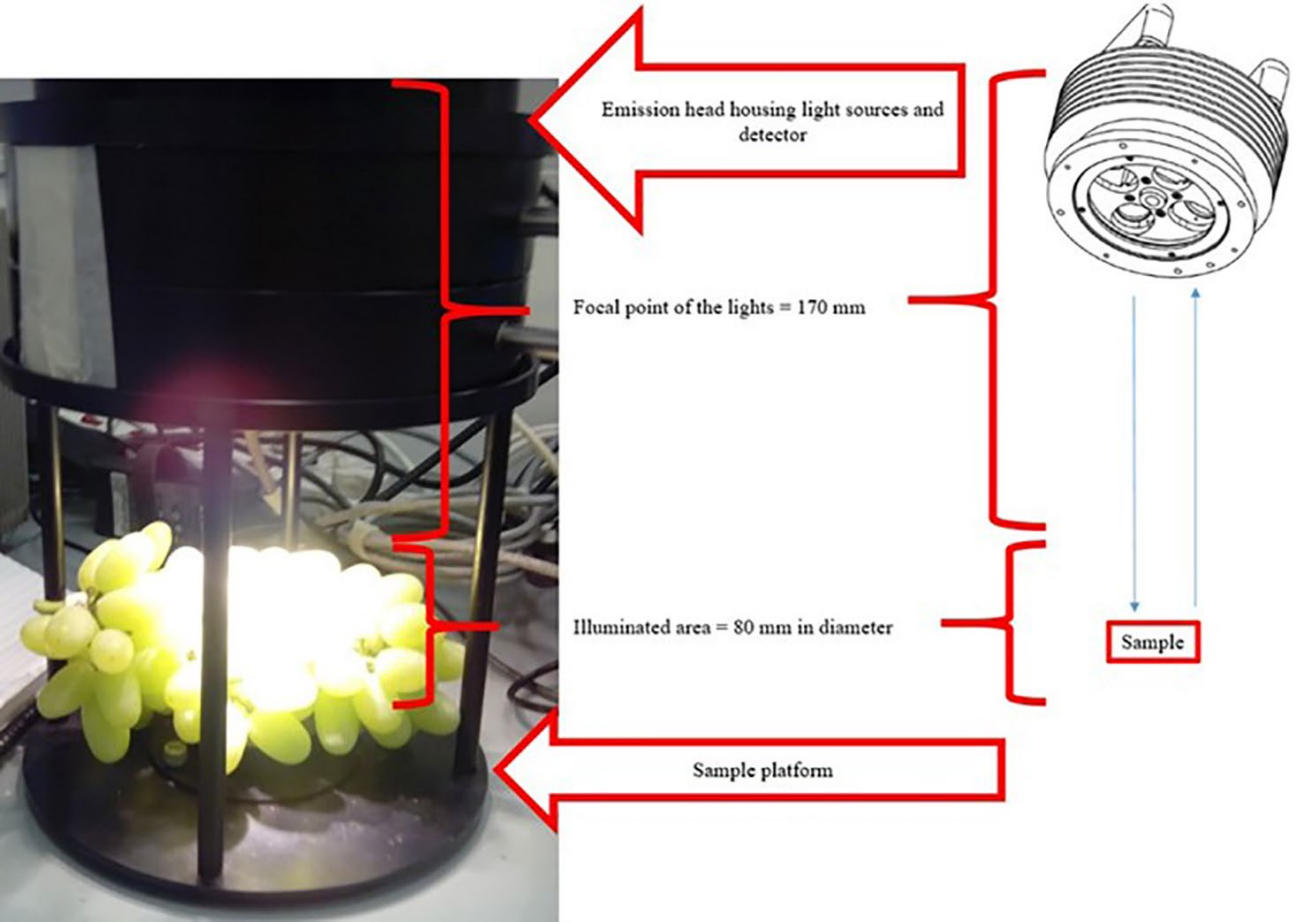
An intact Thompson Seedless table grape bunch scanned contactless with the MATRIX-F NIR spectrometer. Important parts of the instrument are also illustrated.

The laboratory measurement setup was designed so that diffuse reflectance FT-NIR spectra of intact table grape bunches were obtained in a contactless mode by using the MATRIX-F FT-NIR spectrometer connected *via* a fibre optic cable (1 m) to a NIR emission head (Bruker Optics, Ettlingen, Germany), as shown in **Figure 2**. Each bunch was placed on the sample platform directly below four air-cooled tungsten NIR light sources (12 V, 5 W each) housed in the emission head (230 mm diameter, 185 mm height), and scanned individually. Upon illumination of the grapes, the diffuse reflected light was collected and guided back to the spectrometer by the optic cable. The focal point of the lights was 170 mm and the area illuminated on bunches was 80 mm in diameter. The detecting emission head also housed a sensitive, thermoelectric cooled, and temperature-controlled InGaAs diode detector. The scanning procedure per sample took 40 s in which time 32 repeat scans (resolution, 2 cm^-1^; scanner velocity, 10 kHz) were collected in the wavenumber range 800 to 2,500 nm (12,000 to 4,000 cm-1), and averaged into a single absorbance spectrum using OPUS software (OPUS version 7.2 Bruker Optics, Ettlingen, Germany). OPUS works by default in wavenumbers thus 12,000 to 4,000 cm^-1^. Each spectrum consisted of 1,801 data points. A background spectrum was collected using a spectralon in the same way prior to scanning the grape bunches and at hourly intervals during operation of the spectrometer. The spectralon is situated on the sample platform and is covered with a black lid when the sample is being scanned. The Log (1/R) transformed absorbance spectra were processed using OPUS and saved after the spectral acquisition. Each bunch was scanned on two opposite sides, denoted top or bottom, respectively, by turning the bunch manually.

### Reference Measurements

A sampling of twenty grape berries (ten each bunch side - the top and bottom sides) from within the focus area of the NIR light sources (**Figure 2**) was done after the spectra of bunches were generated. Free flowing juice was collected by crushing the berries by hand, for 1 min in a plastic bag, followed by filtration using cheesecloth. TSS of the juice was determined using a handheld digital refractometer (ATAGA Palete Digital Refractometer PR-32 Alpha, Tokyo, Japan). TA and pH were determined with a TIM 865 Titration Manager (Radiometer Analytical, Villeurbanne Cedex, France) automatic titrator. The TSS/TA ratio was calculated by dividing the TSS value of each juice sample by that of the percentage TA (°Brix ÷ %Acid) (Jayasena and Cameron, 2008). BrimA was calculated as TSS – *k* x TA. The constant *k* shows that the tongue is more sensitive to acid than it is to sugar. Due to different fruit containing different ratios of acids and sugars the *k* value range from 2 to 10. A *k* value of 5 is suggested for table grapes and was accordingly used in this study (Jordan et al., 2001).

The standard error of laboratory (SEL) for respectively, TSS ( ± 0.03), TA ( ± 0.05), and pH ( ± 0.20) were based on those reported by the Wine Analytical Laboratory of the Agricultural Research Council, Infruitec-Nietvoorbij in Stellenbosch, South Africa where the samples were analyzed. Certified standards for each parameter were tested daily in triplicate. SEL was calculated as the average of the difference between the true value of the certified standard and the measured result (triplicate measurements). Grape samples were analysed once.

### Data Analysis

To investigate the relationship between the spectral information of the intact bunches and the content of TSS, TA, TSS/TA ratio, pH, and BrimA, PLS regression was implemented in the R statistical environment (R Core Team, 2016) using the “pls” package (Mevik et al., 2016). PLS is a bilinear modelling strategy (Naes et al., 2004) which was used to find the correlation between the spectra taken of the intact table grape bunches and the reference values that was obtained for the maturity parameters TSS, TA, TSS/TA ratio, pH, and BrimA. The data matrix, therefore, consisted of a set of independent X variables (NIR spectral data) and five dependent Y variables TSS, TA, TSS/TA ratio, pH, and BrimA.

Two strategies were used to design calibration and validation sample sets. In Strategy 1, as can be seen in **Figure 1**, a model was created with data from one year (2016) and tested on data from another year (2017). In Strategy 2, the calibration set and the validation sample sets consisted of randomly selected data from both years combined (2016 and 2017). In Strategy 1, the 2016 data (n = 267) was used as the training set and the 2017 data (n = 71) was used as the test set. In Strategy 2, the data sets for 2016 and 2017 were combined (n = 338) and randomly divided into two sub-data sets, i.e., the training set containing 2/3 of the data (n = 204) and testing set containing 1/3 of the total data set (n = 134) for each parameter. A full cross-validation process was applied to build the PLS regression models using the training data set for each parameter

The regression models were evaluated using the coefficient of determination (R^2^) and the Root Mean-Square Error of Calibration (RMSEC) or Validation (RMSECV when cross validation is used and RMSEP when test set validation is used). The R^2^ value, which represents the proportion of explained variance of the response variable in the calibration set (R^2^_c_) or validation set (R^2^_cv_ or r^2^ when cross validation is used and R^2^_p_ when test set validation is used). This value needs to be as high as possible for a good model. It differs from the correlation coefficient (r) which only shows how strong the relationship between two variables are (Taylor, 1990) and R^2^ is a multiple of it (Nagelkerke, 1991). RMSECV is the term indicating the prediction error of the model and the RMSEP value gives the average expected uncertainty for predictions of future samples and both needs to be as close as possible to zero (Brown et al., 2005; Saeys et al., 2005; Esbensen, 2006). The residual prediction deviation (RPD) value is defined as the ratio of the standard deviation of the reference data of the validation set to the standard error of prediction and gives some indication of the efficiency of a calibration (Williams and Norris, 2001). The RPD value has to be between 1.5 and 2 for the model to discriminate low from high values of the response variable; a value between 2 and 2.5 to indicate that course quantitative predictions are possible, and a value between 2.5 and 3 or above to show good and excellent prediction accuracy (Saeys et al., 2005). The standard error of calibration (SEC); standard error of performance (SEP); limit control for SEP (LC_SEP); and limit control for bias (LC_bias) were also calculated. The SEC and SEP, as well as the control limits, also have to be as close as possible to zero to give good working models.

Furthermore, the original data (no spectral preprocessing), as well as five spectral preprocessing techniques, were evaluated for each parameter when the models were built. These were baseline correction, multiplicative scattering correction (MSC) perhaps the most commonly used spectral preprocessing technique followed by standard normal variate (SNV) (Rinnan et al., 2009). These first three are used to correct for any shift that might have occurred in the baseline of the samples and in that way minimize the inconsistency between the samples because of light scatter (Rinnan et al., 2009). In order to enhance the signal to noise ratio, the moving window smoothing (MWS) method is used. This is the standard and easiest one and makes use of a function that smoothes the original data by computing a moving average on a fixed-size spectral window. Before the average can be computed, points outside the spectral window are determined by second-order polynomial extrapolation on both ends of the spectrum (Chau et al., 2004). Savitzky-Golay first derivative (SG1d) also uses smoothing of the spectra before computing the derivative. This is to minimize the negative influence that conventional fixed-difference derivatives would have on the signal-to-noise ratio (Rinnan et al., 2009). A combination of each of the last three spectral preprocessing techniques were used in combination with MSC, i.e., MSW+MSC, SNV+MSC, and SG1d+MSC.

## Results and Discussion

### Intact Bunch Spectral Features

**Figure 3:**
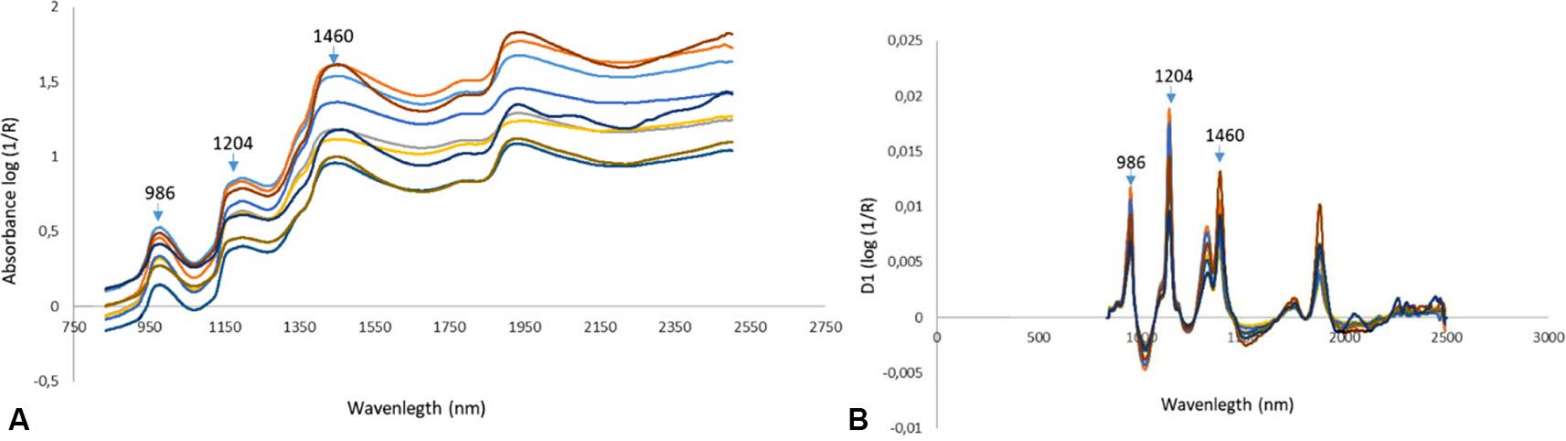
The log (1/R) spectra of intact bunches **(A)** and spectra of intact bunches after Savitzky-Golay First Derivative (SG1d) spectral preprocessing was applied **(B)**.

In **Figure 3**, the characteristic log (1/R) spectra of intact bunches (A) and the spectral preprocessed spectra (B) are displayed. Similarly, as in González-Caballero et al. (2010), the first derivative of the spectra was taken and the effect can clearly be seen through the overlapping absorption bands being separated and absorbance peaks being displayed more clearly (B).

### Reference Data Statistics

A large portion of the soluble solids in grapes is sugars that account for more than 90% of TSS at harvest (Muñoz-Robredo et al., 2011). Kliewer (1967) found that the range of TSS in mature grapes varied widely from 13.7 to 31.5°Brix. **Table 3** shows the statistical analysis of training sample sets of 2016 and 2017 respectively for all the parameters (Strategy 1) and **Table 4** shows them for the training set and the testing set when the two years are combined (Strategy 2). In Strategy 2, the training set contains two-thirds of the data (n = 204) and the testing set contains one-third of the data (n = 137). The minimum value was 10.18°Brix in 2016 and 6.58°Brix in 2017. This was exceptionally low particularly in 2017 given that the intended TSS that the grapes were to be harvested at was 14.0°Brix for Prime Seedless and 16.0°Brix for Regal Seedless and Thompson Seedless according to the standards and requirements regarding control of the export of table grapes (ACT No. 119 OF 1990 of South Africa). However, when the mean (17.59°Brix in 2016 and 15.62°Brix) as well as the range values (14.22°Brix in 2016 and 15.60°Brix in 2017) are considered, they seem to be on par with the standards. Both the standard deviations (SD) and coefficients of variation (CV) values were higher in 2016 (2.37 and 0.13, respectively) compared to 2017 (3.75 and 0.24) as shown in **Table 3**. The trend repeats in **Table 4** when the combined two years and the training and testing sets selection are random. Harvesting of the grapes were at two stages in both years. The second harvest being at a higher TSS level shown by the maximum values contained in **Table 3** (24.40°Brix in 2016 and 22.18°Brix in 2017). This might explain the high coefficient of variation (CV) values as was the case when González-Caballero et al. (2010) also harvested the grapes in their study over different ripening periods.

**Table 3:** Statistical analysis of sample sets for the table grape quality parameters TSS, TA, TSS/TA Ratio, pH and BrimA under study collected in the 2016 and 2017 harvesting seasons to incorporate seasonal changes.

Training Statistic	2016					2017				
Parameter	TSS^a^	TA^b^	TSS/TA Ratio	pH	BrimA	TSS	TA	TSS/TA Ratio	pH	BrimA
N	267	267	267	267	267	71	71	71	71	71
Mean	17.59	4.67	39.24	3.78	5.82	15.62	6.15	29.01	3.78	12.55
Median	17.54	4.40	38.66	3.77	5.88	15.70	5.55	27.67	3.74	12.45
Min^c^	10.18	2.89	15.08	3.31	2.63	6.58	2.97	6.93	3.36	1.83
Max^d^	24.40	7.62	64.28	4.07	10.43	22.18	10.99	66.14	4.29	19.58
Range	14.22	4.73	49.20	0.76	7.80	15.60	8.02	59.21	0.93	17.75
Standard Deviation	2.37	0.90	9.66	0.14	1.35	3.75	2.05	13.45	0.22	4.20
Coefficient of Variation	0.13	0.19	0.25	0.04	0.23	0.24	0.33	0.46	0.06	0.33

^a^Total soluble solids, ^b^Titratable acidity, ^c^Minimum, ^d^Maximum.

**Table 4:** Statistical analysis of randomly selected training (two thirds of data) and test (one third of data) sets for the combined 2016 and 2017 data sets of the table grape quality parameters TSS, TA, TSS/TA Ratio, pH, and BrimA under study.

Training Statistic	Training set					Testing set				
Parameter	TSS^a^	TA^b^	TSS/TA Ratio	pH	BrimA	TSS	TA	TSS/TA Ratio	pH	BrimA
N	204.00	204.00	204.00	204.00	204.00	134.00	134.00	134.00	134.00	134.00
Mean	17.07	4.99	36.89	3.78	7.11	17.17	4.89	36.57	3.78	7.18
Median	17.45	4.62	37.40	3.77	6.23	17.40	4.64	37.18	3.77	6.23
Min^c^	6.58	2.89	6.93	3.31	2.63	6.58	2.89	6.93	3.34	2.63
Max^d^	24.40	10.99	66.14	4.29	19.32	22.96	10.28	61.92	4.29	18.63
Range	17.82	8.10	59.21	0.98	16.69	16.38	7.39	54.99	0.95	16.00
Standard Deviation	2.94	1.41	11.47	0.16	3.35	2.94	1.13	11.21	0.16	3.49
Coefficient of Variation	0.17	0.28	0.31	0.04	0.47	0.17	0.23	0.31	0.04	0.49

^a^Total soluble solids, ^b^Titratable acidity, ^c^Minimum, ^d^Maximum.

Grapes also contain significant amounts of organic acids. These are very important components of grape juice, since they are responsible for the tart taste and have a marked influence on juice stability, color, and pH (Fahmi et al., 2012). During berry development, TA usually decreases as TSS increases. The juice pH is a measure of the hydrogen ion concentration in the berry generally related to juice acidity. Although there is no direct relationship between TA and pH, higher acid levels in fruit are often associated with lower pH values and vice versa as can be seen in **Table 3** especially in terms of the maximum values in 2016 (TA = 7.62 g/L and pH = 4,07) and 2017 (TA = 10.99 g/L and pH = 4.29). The juice pH of Thompson Seedless grapes usually ranges between 3.5 and 3.9 at harvest. Vial et al. (2005) obtained a mean of 3.46 in the experiments they conducted and Fahmi et al. (2012) one of 4.05 in theirs. This falls within the range obtained in 2016 (4.73 g/L) but not in 2017 (8.02 g/L) and in Strategy 2 (8.10 g/L and 7.39 g/L respectively for the training and testing sets). This is due to the higher minimum (2.97 g/L) and maximum (10.99 g/L) values that were obtained in 2017. This highlights a very significant effect that seasons can have on the development of grapes as could also be clearly seen in the minimum and maximum values of TSS which were lower in 2017 (6.58°Brix and 22.18°Brix) then in 2016 (10.18°Brix and 24.40°Brix). The temperature difference between these two seasons (**Table 2**) probably played a role with the average maximum daily temperatures being mostly higher and the average minimum temperature being mostly lower during the ripening and harvest period. All metabolic processes in plants such as photosynthesis responsible for carbohydrate manufacturing (TSS) are temperature dependent (Berry and Björkman, 1980).

According to the South African standards and requirements regarding control of the export of table grapes (ACT No. 119 OF 1990), the acceptable TSS/TA values for table grapes are 22 for Prime Seedless, 24 for Regal Seedless and 21 for Thompson Seedless. When the minimum values are considered, they are below these and the maximum values are above these, with the mean value being much higher in 2016 (39.24) than in 2017 (29.01) and the range the other way around for the two years 49.20 in 2016 and 59.21 in 2017. Jayasena and Cameron (2008) obtained values of up to 40 for Crimson Seedless in their study similar to the mean of this study in 2016.

BrimA is not a widely used parameter for table grapes and has only thus far been proposed by Jordan et al. (2001) and evaluated by Jayasena and Cameron (2008) who found that it could not give better predictive results for the sensory qualities of Crimson Seedless table grapes than what TSS/TA could. However, BrimA has been reported as a valuable maturity index and quality parameter for a wide range of fruits including mango (Wongkhot et al., 2012), pomegranate (Fawole and Opara, 2013; Arendse et al., 2014), citrus (Ncama et al., 2017), and grapefruit (Olarewaju et al., 2018). The acceptable minimum and maximum values as well as median and ranges is, therefore, still to be established and may differ from the ones achieved in **Tables 3** and **Table 4** when other table grape cultivars are added.

### Performance of Calibration Models

Daniels et al. (2018) showed that the best calibration models were obtained when the average spectra of table grape bunches were used to construct the respective models. **Table 5** shows the results of the calibration models for TSS, TA, TSS/TA ratio, pH, and BrimA (Strategy 1). **Table 6** shows the results for the same parameters but built using Strategy 2. Construction of models was with data of the original spectra as well as the baseline corrected spectra, but only results of the models with the original spectra are shown since they always performed better. The best model was selected in terms of which spectral preprocessing technique or combination of techniques gave the most appropriate values for the statistics used to measure the strength of the model.

**Table 5:** Performance of Partial Least Squares (PLS) models for table grape quality parameters using 2016 data as the training set (n = 267) and 2017 as the testing set (n = 71). Also shown is the preprocessing techniques that gave the best model.

Parameter	TSS^a^	TA^b^	TSS/TA ratio	pH	BrimA
Spectral preprocessing technique	SNV^c^	SNV	SNV	SG_1d_^d^	MSC^e^
LVs^f^	20	18	20	4	11
R^2^_c_^g^	0.92	0.66	0.67	0.31	0.27
R^2^_cv_^h^	0.83	0.32	0.32	0.16	0.06
R^2^_p_^i^	0.71	0.16	0.14	0.07	0.09
SEC^j^	0.68	0.52	5.50	0.12	0.12
SEP^k^	2.09	1.89	12.55	0.21	0.21
LC_SEP^l^	0.88	0.67	7.15	0.15	0.16
LC_bias^m^	0.41	0.31	3.30	0.07	0.07
RMSEC^n^	0.68	0.52	5.49	0.12	0.12
RMSEP°	2.18	2.51	19.86	0.21	0.21
RPD_c_^p^	3.51	1.73	1.76	1.21	1.17
RPD_p_^q^	1.09	0.36	0.49	0.68	0.67

^a^Total soluble solids, ^b^Titratable acidity, ^c^Standard Normal Variate, ^d^Savitzky-Golay first derivative, ^e^Multiplicative scatter correction, ^f^Latent variables, ^g^Coefficient of determination for the calibration set, ^h^Coefficient of determination for cross validation, ^i^Coefficient of determination for prediction, ^j^Standard error of calibration, ^k^Standard error of performance, ^l^Limit control for SEP (LC_SEP), ^m^Limit control for bias, ^n^Root mean square error of calibration), °Root mean square error for prediction, ^p^Residual prediction deviation for calibration, ^q^Residual prediction deviation for prediction.

**Table 6:** Performance of Partial Least Squares (PLS) models for table grapes quality parameters of randomly selected training (n = 204) and test set (n = 134) samples of the combined 2016 and 2017 data. Also shown is the preprocessing techniques that gave the best model.

Parameter	TSS^a^	TA^b^	TSS/TA ratio	pH	BrimA
Preprocessing strategy	MSW^c^+MSC^d^	No spectral pre-processing	SG_1d_^e^	SG_1d_	MSW+MSC
LVs^f^	21	23	5	7	24
R^2^_c_^g^	0.95	0.80	0.76	0.66	0.95
R^2^_cv_^h^	0.88	0.47	0.61	0.21	0.78
R^2^_p_^i^	0.71	0.33	0.57	0.28	0.77
Sec^j^	0.61	0.67	5.31	0.09	0.75
Sep^k^	1.50	1.08	7.86	0.14	1.81
LC_Sep^l^	0.79	0.87	6.91	0.12	0.98
LC_bias^m^	0.36	0.40	3.19	0.06	0.45
RMSEC^n^	0.61	0.67	5.30	0.09	0.75
RMSEP°	1.52	1.09	7.83	0.14	1.80
RPD_c_^p^	4.72	2.26	2.05	1.72	4.57
RPD_p_^q^	1.89	1.38	1.39	1.13	1.90

^a^Total soluble solids, ^b^Titratable acidity, ^c^Moving smoothing windos, ^d^Multiplicative scatter correction, ^e^Savitzky-Golay first derivative, ^f^Latent variables, ^g^Coefficient of determination for the calibration set, ^h^Coefficient of determination for cross validation, ^i^Coefficient of determination for prediction, ^j^Standard error of calibration, ^k^Standard error of performance, ^l^Limit control for SEP (LC_SEP), ^m^Limit control for bias, ^n^Root mean square error of calibration), °Root mean square error for prediction, ^p^Residual prediction deviation for calibration, ^q^Residual prediction deviation for prediction.

### TSS, TA, TSS/TA Ratio, pH, and BrimA

The best predictive results for TSS was obtained with MSW+MSC as spectral preprocessing technique with Strategy 2. When González-Caballero et al. (2010) scanned whole wine grape bunches to assess the SSC the authors obtained value of 0.57 and higher SEP, LC_SEP, and LC_bias values of 1.63, 0.62, and 1.35, respectively. Cao et al. (2010) found r and RMSEP of 0.91 and 0.96 for SSC and similarly did Baiano et al. (2012) and Omar (2013) who found R^2^ and RMSE of 0.94 and 0.06 and 0.95 and 0.18, respectively. The R^2^ value is higher and the RMSE values lower because they scanned single table grape berries and not intact table grape bunches as in this study. This is also clearly illustrated in the study of Parpinello et al. (2013) that found values for r^2^ = 0.85, RMSECV = 1.08, SECV = 1.08, and RPD = 2.6 when using cross-validation instead of test set validation. The data in all the other experiments were also collected from a single year and not over two years as in this study.

The best model for TA was also achieved with Strategy 2 when SNV was used as spectral preprocessing technique. Baiano et al. (2012) found the R^2^ and RMSE to be 0.95 and 0.06 for TA using 5 LVs for the construction of their models.

TSS/TA ratio gave the best model with Strategy 2 when SG1d was used as spectral preprocessing techniques.

González-Caballero et al. (2010) also scanned intact bunches for amongst others SSC, TA, and pH, but it was of wine grapes and the physiology of wine grape bunches are different from those of table grapes. Wine grape bunches and berries are much smaller than those of table grapes and the berries are also situated much closer together (more compact) than table grape bunches. Table grape bunches tend to be looser due to not only having longer pedicels, but also due to the bunch preparation that were done on them such as thinning and removal of small and uneven berries before harvesting.

The best model for pH was achieved with Strategy 2 when SG1d was used as spectral preprocessing technique. Cao et al. (2010) found r and RMSEP were 0.98 and 0.13 for pH, and 0.91 and 0.96 for SSC respectively in the prediction set. Baiano et al. (2012) found the pH validation values for R^2^ and RMSE to be 0.80 and 0.06 and Omar (2013) found R^2^ = 0.763 and RMSE = 0.11. Gonzalez-Caballero et al. (2010) made use of test set validation and found the best predictive values for pH (r^2^ = 0.51, SEP = 0.19, LC_BIAS = 0.06, LC_SEP = 0.13). These values were similar to those in this study except the r^2^ that was lower (0.28).

BrimA gave the best model with Strategy 2 when MSC was used as spectral preprocessing technique. The RPD values obtained for the BrimA model was the highest overall and the only one other than that for TSS indicating that the model is able to discriminate low from high values of the response variable (Saeys et al., 2005). For the rest of the parameters, TA, TSS/TA ratio, and pH, this value indicates that the models are not ready yet to be used for discrimination purposes since it is below 1.5 (Saeys et al., 2005). RPD was rarely reported in the published literature as a statistic to evaluate the strength of calibration models for the parameters of interest. Parpinello et al. (2013) reported a RPD value of 2.6 for SSC for single table grape berries. In the present work, on intact bunches, a significantly higher RPD values for the calibration stage (RPD_c_^p^) were obtained for TSS (4.72 in **Table 6**). In one study, on intact wine grape bunches, González-Caballero et al. (2010) reported RPD values for SSC ranging from 2.92 to 3.18 depending on the spectral range used to establish the calibration models. A comparison of RPD values obtained for TA and pH showed that the results obtained in the present study were comparable to those reported by González-Caballero et al. (2010). The R^2^ values obtained in the present study for BrimA were considerably better than those found for the TSS/TA ratio. This was also the case in the research work of Jordan et al. (2001). The R^2^ values for BrimA were mostly above 70% where those for the TSS/TA ratio were always just above 60%.

The major difference in the results of the two different calibration sample selection strategies was the much higher RMSEP values that were obtained for all the parameters, except BrimA with Strategy 1. Low RPD values were also obtained with Strategy 1 (**Tables 5** and **6**). A major contributor towards this difference may have been the higher maximum values for all the parameters, except TSS that was present in the 2017 dataset that was used for validation. Samples with similar or higher values should have been present in the calibration dataset (2016) as well. For TSS the minimum value of 2017 again was not present in the calibration set and similar samples would thus not have been able to be predicted.

The SEL values were in all instances much lower than the RMSEC and RMSEP values obtained with the models, highlighting the fact that the accuracy of models constructed using data captured through NIR spectroscopy can never be as good as the standard reference method used. These results underscore the importance of updating calibration models with samples from future harvests (Guthrie et al., 2005) as well as the use of different calibration ranges as was done in González-Caballero et al. (2010).

### Effect of Spectral Preprocessing Techniques

All the spectral preprocessing techniques and combination with MSC had various effects on the results obtained for each parameter (**Tables 5** and **6**). Parpinello et al. (2013) also evaluated five spectral preprocessing techniques but does not show the effect each specific spectral preprocessing technique had on each model, but states that a combination of mean normalization (MN)+MSC delivered the best model for SSC when discriminant analysis (DA) was performed. Baiano et al. (2012) also evaluated second derivatives. They, however, found that not any of the spectral preprocessing technique could create a better model than the original spectra. Cao et al. (2010) just made use of averaging and not any specific spectral preprocessing technique. It is however clear from the results shown here that a specific spectral preprocessing technique will not always deliver all the desired statistical values that constitute for a good model. Thus, one spectral preprocessing technique or combination with another, for example, SNV or MSW alone or each combined with MSC will not always deliver the highest R^2^ and RPD values and lowest SEP, RMSEP, and control limits for a parameter as desired. This can most probably be contributed to the different regions or areas of the spectrum that is highly associated with the chemistry of each parameter, which was not evaluated in this study. In Poblete-Echeverría et al. (2018), however, a decrease in predictive accuracy was obtained with variable selection in both the artificial neural network (ANN) and PLS models, but a good result was obtained with spectral preprocessing applied in the final PLS model.

### Latent Variables

**Figure 4:**
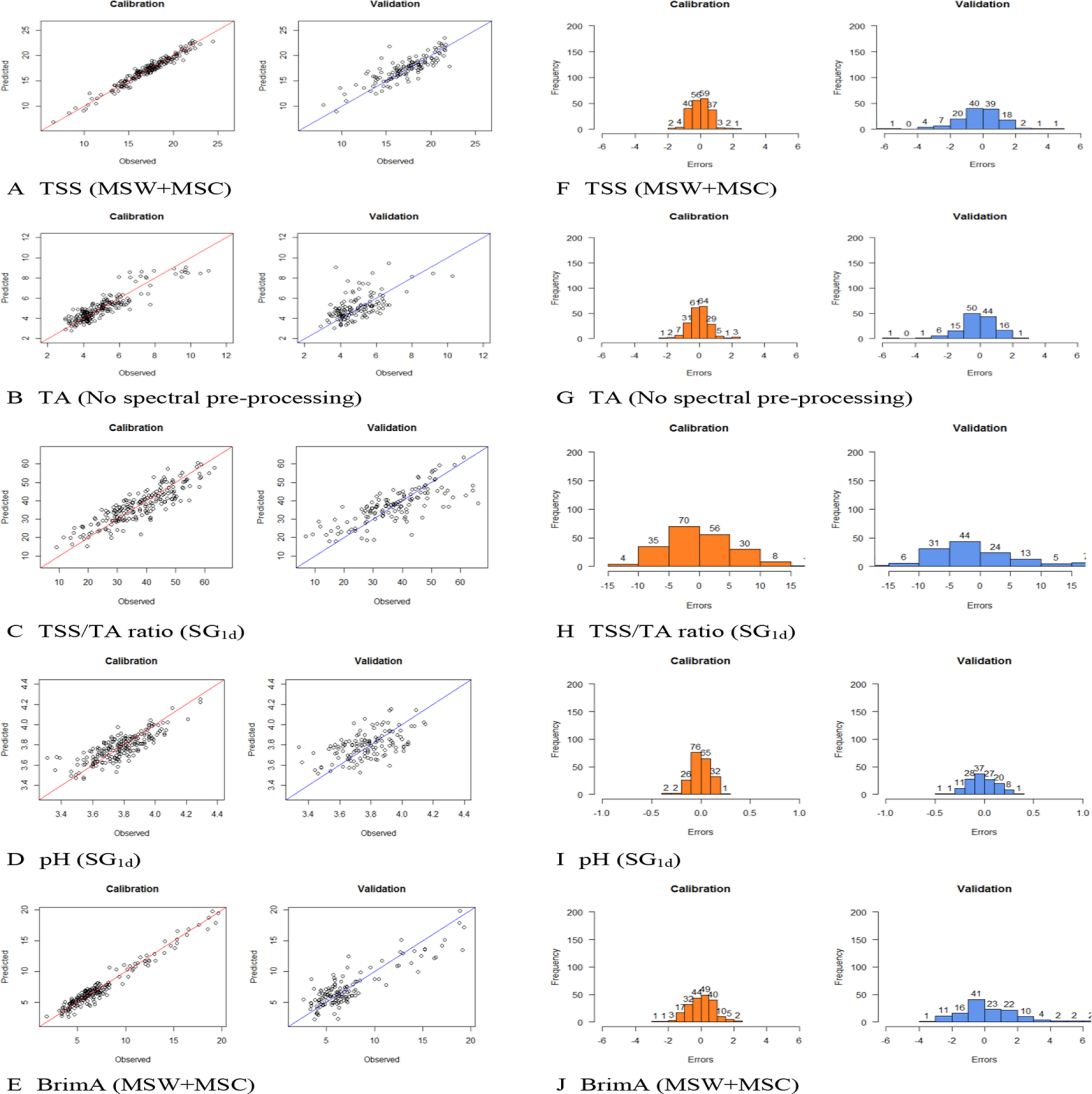
Calibration and validation plots of the models obtained for the five parameters and the spectral preprocessing methods applied to the raw spectra during the construction process; **(A)** total soluble solids (TSS), **(B)** titratable acidity (TA), **(C)** TSS/TA ratio, **(D)** pH, and **(E)** BrimA as well as the distribution of the errors obtained for each model **(F)** TSS, **(G)** TA, **(H)** TSS/TA, **(I)** pH, and **(J)** BrimA.

The number of LVs used to construct the best model for the parameters varied from as little as four for pH and as high as 24 for BrimA. The optimum number of principal components (latent variables) in case of PLS seems to be three at the lowest level of residual validation variance (Jha et al., 2006). A relatively low number of LVs are generally desirable to avoid modelling noise signals (Fernández-Novales et al., 2009). This especially not to compromise the robustness of the models for future predictions. The lowest number of LVs should thus be that which always gave the lowest error as to not make the models too complex by using more factors that are necessary (Rinnan et al., 2009). This is, however, not always possible as can be seen in this study. Parpinello et al. (2013) obtained the best model with 17 LVs for SSC when monitored in each berry of intact bunches in order to evaluate intra-bunch distribution and variability. A number that is comparable to the numbers used here. Baiano et al. (2012) used nine, seven, and nine for SCC, pH, and TA respectively, which are lower than the numbers used here to achieve the lowest error. Only when SG1d was used were such low number of LVs used, but they did not give the lowest errors. **Figure 4** shows the calibration and validation plots of the models obtained for the five parameters and the spectral preprocessing strategy applied to the raw spectra during the construction process as well as the distribution of the errors obtained with each model. It can be seen in the calibration plots that the samples are not always spread evenly along the regression line in the validation plots as they are in the calibration plots. The same way that the frequency and the spread of the errors are, not the same in the calibration and validation bar plots. This shows clearly that the models should thus not only be evaluated on the numerical values of the statistics but also on the visual distribution of the samples and/or errors.

### Calibration Ranges

The better prediction statistics obtained for TSS are due to not only the higher concentration level of TSS present in the grapes, but also due to the wide range over which it spreads (6.58–24.40). The values of TA and pH spreads over a very narrow range, 2.89–10.99 g/L for TA and 3.31–4.29 for pH making the construction of a proper calibration model extremely difficult as can be seen in **Figure 4**. Moreover, given that NIR spectra contain overtones and combinations derived from fundamentals which appear in the infrared region (Skoog et al., 1997) and measures the vibrational transitions of molecular bonds, such as the O-H bonds in water, and bonds such as C-N, N-H, and C = O, characteristic to organic matter (Rinnan and Rinnan, 2007). TSS is predominantly consisting of water and sugar, making the creation of a good calibration easier unlike pH that cannot actually be measured directly seeing that the activity of single ion (H+) is involved (Covington et al., 1985). Its accuracy, therefore, depends on the operation used to measure it, usually in a liquid state, as done during the reference measurements in this experiment and not nondestructively and intact as set out in this experiment.

Due to the fact that table grapes mainly consist of water like many other fruit and vegetables, NIR spectra are complex and are dominated by the water peaks (Nicolaï et al., 2007) in the wavelength ranges from 1400–1440 nm and 1900 to 1950 nm (Bünning-Pfaue, 2003), as can be seen in **Figure 3A**. Since grape sugars are dissolved in water, the wavelengths that are strongly associated with the O-H and C-H first and second overtones associated with sugar are usually masked in those areas (Cozzolino et al., 2006; Dambergs et al., 2006). First derivative of the spectra using the Savitzky-Golay algorithm as was done in this study to enhance these peaks (**Figure 3B**).

**Figure 5:**
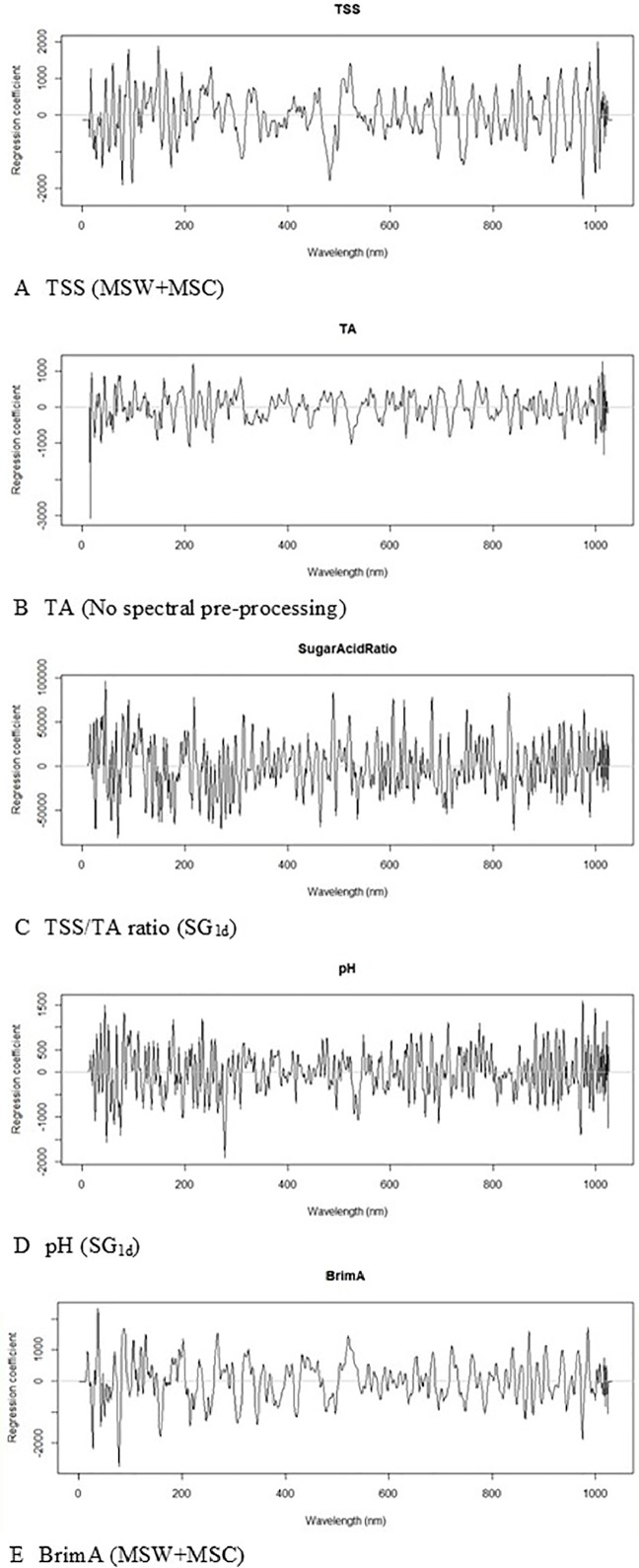
Partial Least Squares (PLS) beta-coefficient plots obtained during the calibration construction process of **(A)** total soluble solids (TSS), **(B)** titratable acidity (TA), **(C)** TSS/TA ratio, **(D)** pH, and **(E)** BrimA.

The PLS beta coefficient is also a very good indication of which wavelengths play a dominant role in the calibration model (Maghirang et al., 2003: Nagle et al., 2010). In **Figure 5**, the regression coefficients for all the best models for the wavelength region up to 1000 nm are shown and the peaks at 950 nm and 980 nm are strongly associated with TSS and that at 980 nm for pH as in González-Caballero et al. (2010). Giovenzana et al. (2014) identified 670 nm, 730 nm, and 780 nm as being highly associated with TSS in wine grapes. It is not uncommon to use the entire NIR region (Jarén et al., 2001) during calibration as was done in this study, although the use of specific regions has also been reported (Herrera et al., 2003; Bellincontro et al., 2011). The regression plots for TA highlighted the difficulty of assigning a specific wavelength to this parameter, since it is made up of several different acids, and likewise the TSS/TA ratio and BrimA parameters which are calculated from the TSS and TA values.

It is important to note when comparing the results obtained here to those on berry experiments of the work of other authors (Baiano et al., 2012; Omar, 2013; Parpinello et al., 2013) that the focus area of the light source on their samples was short, and not 17 cm as in this experiment. It is thus remarkable that the spectra could capture enough of the information in the grape bunches. This not only because of the heterogeneous nature of grape bunches which consists of a rachis berries, and pedicels, but also due the usually low penetration depth of NIR light into a sample.

## Conclusions

The development of models with RPD values which can discriminate between high and low values of TSS, TA, and TSS/TA ratio together with low RMSEP values, can greatly help minimize the losses suffered by producers due to the incorrect determination and classification of grapes for the export market based on these parameters.

Another implication of these results for the table grape industry is much quicker decisions taken over the quality of the grapes either using one of the parameters or all of them collectively to determine which class and which export markets table grapes should be send to. This especially with the inclusion of BrimA which can now help producers with the sensory quality of table grapes, so they can market them accordingly based on consumers’ palates, e.g., low sweetness-high acidity, neutral, high sweetness-low acidity tasting grapes, etc.

Future work will be to build better models for especially pH and TA. This will be explored through the selection of specific wavelengths strongly associated with these two parameters. When different strategies are used to build NIRS models, sampling should be done in such a way that in the end both the calibration and validation sets contain samples that are represented in each.

## Data Availability Statement

The datasets generated for this study are available on request to the corresponding author.

## Author Contributions

AD, UO, and HN conceptualized the research. AD conducted the experiments. AD, CP-E, and HN made the data analyses. AD, UO, CP-E, and HN wrote the manuscript.

## Funding

This study was supported by the South African Table Grape Industry (SATI) and the Perishable Exports Control Board (PPECB) of South Africa. This work is based on the research supported in part by the national Research Foundation of South Africa (Grant Number: 64813). The opinions, findings, and conclusions or recommendations expressed are those of the author(s) alone, and the NRF accepts no liability whatsoever in this regard.

## Conflict of Interest

The authors declare that the research was conducted in the absence of any commercial or financial relationships that could be construed as a potential conflict of interest.
